# Idiopathic Myointimal Hyperplasia of the Mesenteric Veins: A Case Report and Scoping Review of Previously Reported Cases From Clinical Features to Treatment

**DOI:** 10.3389/fmed.2022.855335

**Published:** 2022-04-13

**Authors:** Hui Li, Hong Shu, Hong Zhang, Mingming Cui, Yuying Gao, Feng Tian

**Affiliations:** ^1^Department of Gastroenterology, Shengjing Hospital of China Medical University, Shenyang, China; ^2^Department of Pathology, Shengjing Hospital of China Medical University, Shenyang, China; ^3^Department of General Surgery, Shengjing Hospital of China Medical University, Shenyang, China; ^4^Department of Radiology, Shengjing Hospital of China Medical University, Shenyang, China

**Keywords:** idiopathic myointimal hyperplasia of the mesenteric veins, scoping review, cronh’s disease, inflammatory bowel disease, ischemic enteritis

## Abstract

Idiopathic myointimal hyperplasia of the mesenteric veins (IMHMV) is a rare and poorly understood disease. It is characterized by non-thrombotic and non-inflammatory occlusion of the mesenteric veins secondary to intimal smooth muscle hyperplasia. The etiology of IMHMV is unknown, and its clinical presentations include abdominal pain, bloody diarrhea, and weight loss. IMHMV is commonly mistaken for inflammatory bowel disease because of the similarity in symptoms and endoscopic findings. Herein, we report the case of a 64-year-old man with IMHMV and present an overview of all reported cases of IMHMV. In this review, we analyzed 70 cases to summarize the etiology, clinical manifestations, and diagnosis of IMHMV and hope to raise clinicians’ awareness of this entity.

## Introduction

Idiopathic myointimal hyperplasia of the mesenteric veins (IMHMV) is a rare cause of chronic intestinal ischemia, which is characterized by venous occlusion resulting from the proliferation of the smooth muscle in the venous intima without thrombosis (1). It was first reported by Genta and Haggitt in 1991, who described four male patients with segmental ischemic colitis that were essentially cured and recurrence-free after bowel resection (1). Since then, 70 cases of IMHMV have been reported.

IMHMV often affects middle-aged and older adults, especially previously healthy men, and the typical presentation is recurrent, progressive abdominal pain accompanied by bloody diarrhea and weight loss. A definitive diagnosis is possible only after surgery, as biopsies are not capable of distinguishing it from other bowel diseases. IMHMV is frequently confused with inflammatory bowel disease (IBD) clinically and poses a diagnostic challenge to clinicians and pathologists (2).

Herein, we describe a case of IMHMV with intestinal obstruction and ileal fistula. We review reported cases of IMHMV in the literature: (1) to summarize its etiology, clinical features, and diagnosis from pathological, endoscopic, and radiological aspects; (2) to compare the difference between IMHMV and Crohn’s disease; (3) to analyze the possible factors (steroid usage) associated with severe complications. We hope this review can help clinicians gain a better understanding of this disease and make a definite diagnosis of IMHMV pre-operation.

## Data and Methodology

### Case Presentation

**FIGURE 1:**
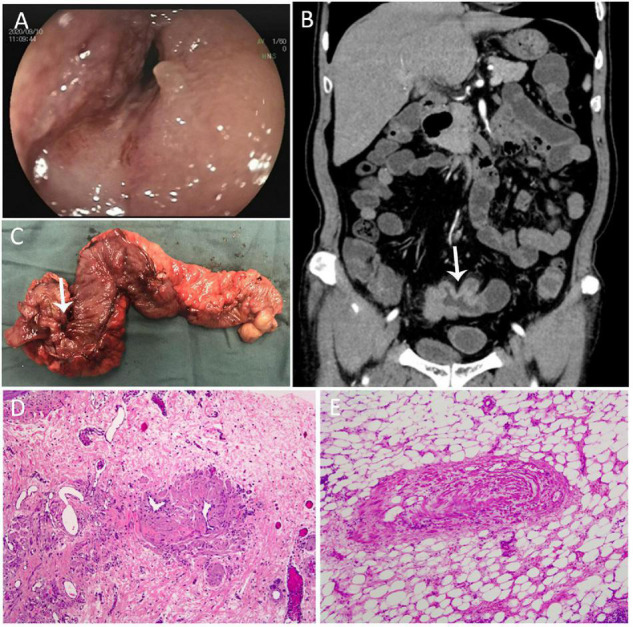
**(A)** Double balloon enteroscopy showed severe mucosal congestion and luminal stenosis with two deep 1.2–1.5 cm-long longitudinal ulcers in the terminal ileum. **(B)** Computed tomography enterography showed a thickened pelvic ileum wall with adherence, and an intestinal fistula was suspected. **(C)** The thickened wall, luminal stricture, and fistula were seen in the resected terminal ileum. **(D)** Histological examination revealed multiple focal congestion, irregular crypts, and pylorus gland metaplasia, and recanalization was observed in some stenotic veins. **(E)** The wall of the medium-caliber mesenteric veins was thickened with proliferated smooth muscle and a narrow lumen.

A 64-year-old, healthy Chinese male patient was admitted to our hospital with chief complaints of abdominal pain, distension, and weight loss for 6 months. Gastroscopy and colonoscopy were performed, and no ulcers or obvious inflammation were found. Blood tests for autoimmune conditions, systemic vasculitis, and thrombophilia yielded normal results. On double-balloon enteroscopy, severe mucosal congestion and luminal stenosis were seen with two deep 1.2–1.5 cm-long longitudinal ulcers in the terminal ileum (Figure 1A). CT enterography revealed a thickened pelvic ileum wall with adherence, and an intestinal fistula was suspected (Figure 1B). Partial ileectomy was performed laparoscopically to further confirm the diagnosis. Two segments of lesions were found with a thickened wall, luminal stricture, and fistula formation in the resected terminal ileum 5 and 20 cm proximal to the cecum (Figure 1C). Histological examination revealed segmental distribution of small intestinal ulcers, mostly in the submucosa. Multiple focal congestion, irregular crypts, and pylorus gland metaplasia were observed without obvious full-thickness inflammation (Figure 1D). The wall of the medium-caliber mesenteric veins was thickened with proliferated smooth muscle and a narrow lumen. Recanalization was observed in some stenotic veins (Figure 1E). Therefore, a diagnosis of IMHMV was made. Postoperative recovery was uneventful, and the patient reported defecating 2–3 times a day over one year of follow-up.

### Literature Review

We performed an extensive review of the literature from 1991 until February 2022 using electronic databases (Medline, EMBASE, Web of Science, PubMed, and The Cochrane Library – CENTRAL). The specific keywords included “Idiopathic myointimal hyperplasia” or “ischemia” AND “mesenteric.”

## Discussion

### Etiology

The etiology of IMHMV remains unclear. The underlying diseases in the reported cases include hypertension, aortic valve replacement, diabetes mellitus, cerebral infarction, abdominal surgery, pulmonary embolism, hyperlipidemia, primary biliary cirrhosis, and renal transplantation (3–11). However, most patients were previously healthy with no significant drug history, and due to the rarity of IMHMV, it is still uncertain whether these underlying diseases correlate with the occurrence of vessel abnormalities.

Another theory that can contribute to IMHMV is the formation of an arteriovenous fistula (AVF). Since the histological features of the veins in IMHMV bear a striking similarity to those of failed cardiac saphenous vein bypass grafts as well as stenosis of AVFs in patients undergoing dialysis (12, 13), the Genta and Haggitt hypothesis states that the formation of AVFs might increase venous blood flow and result in the development of this disease (1). As the sigmoid colon is hypermobile and at risk of volvulus, intermittent torsion or stretching injury to the sigmoid mesocolon might lead to AVF formation, elevated venous pressure, and vascular remodeling (14). However, to the best of our knowledge, no arteriovenous malformation has been identified in any of the reported cases. A retrospective study including 68 non-neoplastic bowel resection specimens found 10 cases with focal IMHMV-like changes in both patients with prior trauma and without prior trauma to the abdomen, with a higher proportion in the former group. The significant association between prior trauma to the resected bowel segment and focal IMHMV might support the trauma hypothesis suggested as a plausible pathomechanism for IMHMV (15). However, since IMHMV is rare with about 70 cases reported till date, it is still uncertain whether IMHMV-like changes are a pathological phenomenon secondary to primary bowel inflammation or trauma or an independent disease entity. Although the hemodynamic theory may explain why such cases are seen in the sigmoid colon of young, healthy, physically fit adults, this hypothesis fails to account for the occurrence of IMHMV in other sites involving the jejunum, ileum, and the entire colon (16). Another study reported a case of a 59-year-old man with a 30-year history of pathologically diagnosed Crohn’s disease (CD). However, IMHMV was diagnosed based on the histopathology of the ileum and colon during the patient’s third surgery (16). Although IMHMV might be suspected as the initial diagnosis or secondary to bowel inflammation, various factors, including immune dysfunction, drugs, and toxins, may contribute to the pathogenesis of the disease (17).

Enterocolic lymphocytic phlebitis (ELP), an enterocolic venous disease similar to IMHMV, is histologically characterized by lymphocytic infiltration into the mural and mesenteric veins (18–21). Previous reports have suggested that IMHMV and ELP may belong to the same disease spectrum (17, 22). Potential overlap between the two disorders is highlighted by a case reported as ELP with histological findings typical of IMHMV but with scattered lymphocytes within the mesenteric veins (23). A case of ELP with prominent myointimal hyperplasia shared characteristics of both ELP and IMHMV, suggesting that they may theoretically represent different stages of the same disease process (24). This is likely to be responsible for cases of small intestine IMHMV, which cannot be explained by motility disorders as in the colon type. However, further studies are needed to confirm this hypothesis.

### Pathological Characteristics

Idiopathic myointimal hyperplasia of the mesenteric veins of the colon is characterized macroscopically by thickened walls, colon stricture, and large and indurated lobules of the epiploic mesenteric fat encroachment on the anti-mesenteric aspect of the colon, which could be considered a key point to distinguish it from CD (16, 22).

Historically, histopathologic characteristics found after surgical resection were considered the only method to definitively diagnose IMHMV. IMHMV mostly involves the thickening of small and medium-sized intramural mesenteric veins, with the hallmark manifestation of intima and media smooth muscle proliferation resulting in luminal occlusion and mucosal ischemic changes (16); intimal thickening is usually circumferential but may occasionally be eccentric without inflammatory infiltration (1). In some cases, intimal thickening resulted in complete or near complete occlusion of the vascular lumen, which was associated with the severity of intestinal inflammation and clinical symptoms. However, the accompanying arteries were completely spared. Elastin staining highlights the elastic laminae present in arteries to better distinguish it from the thickened venous intima (3).

Evidence shows that IMHMV can also affect the mucosa, raising the possibility of endoscopic diagnosis. Although the absence of concrete mucosal histopathological criteria of IMHMV makes the biopsy-based diagnosis a challenge for pathologists, some pathological features may help the preoperative diagnosis of the disease, especially in cases with clinically doubtful IBD. A case report describing the histological findings in a patient with IMHMV noted the presence of thick-walled hyalinized vessels in mucosal biopsies, which were absent in other types of ischemic enterocolitis (14). Wang et al. reported a case preoperatively diagnosed IMHMV based on the detection of an ischemic pattern of mucosal damage, with fibrin deposition and myointimal thickening of the small blood vessels within the lamina propria in biopsies (8). In a case–control study, colonoscopy biopsy samples from seven patients with IMHMV were assessed (22). The presence of clustered, slightly dilated, “arteriolized” capillaries lined by plump endothelial cells and subendothelial fibrin deposits may assist in the diagnosis based on biopsy specimens. Histological features of vessel remodeling were suspected to be secondary to chronic mechanical stress on the mesenteric veins. The increased venous pressure transmitted to the mucosal capillaries led to endothelial injury and resulted in fibrin extravasation (22). These mucosal findings on biopsies were further confirmed by another study, that reported seven cases of IMHMV with a similar mix of ischemic changes and dilated, thick-walled capillaries on pre-resection biopsies. In a few biopsies, thickened submucosal veins were also observed (25). These typical features suggest the possibility of a preoperative diagnosis of IMHMV.

### Clinical Features

**FIGURE 2:**
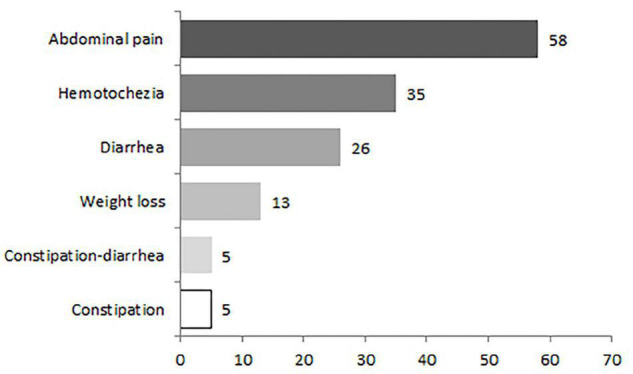
Common clinical symptoms in 70 patients with idiopathic myointimal hyperplasia of the mesenteric veins (IMHMV).

Patients with IMHMV may present with non-specific symptoms, including abdominal pain (82.9%, 58/70), hematochezia (50%, 35/70), diarrhea (37.1%, 26/70), weight loss (18.6%, 13/70), constipation alternating diarrhea (7.1%, 5/70), and constipation (7.1%, 5/70), resulting in a 52.9% IBD misdiagnosis rate (37/70) (Figure 2). The clinical course is usually chronic, involving weeks or months. However, there were eight cases with acute onset of the disease documented in the literature with complaints of watery diarrhea and abdominal pain (7, 11, 17, 25–29). The mean age at diagnosis was 58 (range, 21–83) years. Men were more likely to be affected than women by a 4.8:1 ratio (58/12).

**FIGURE 3:**
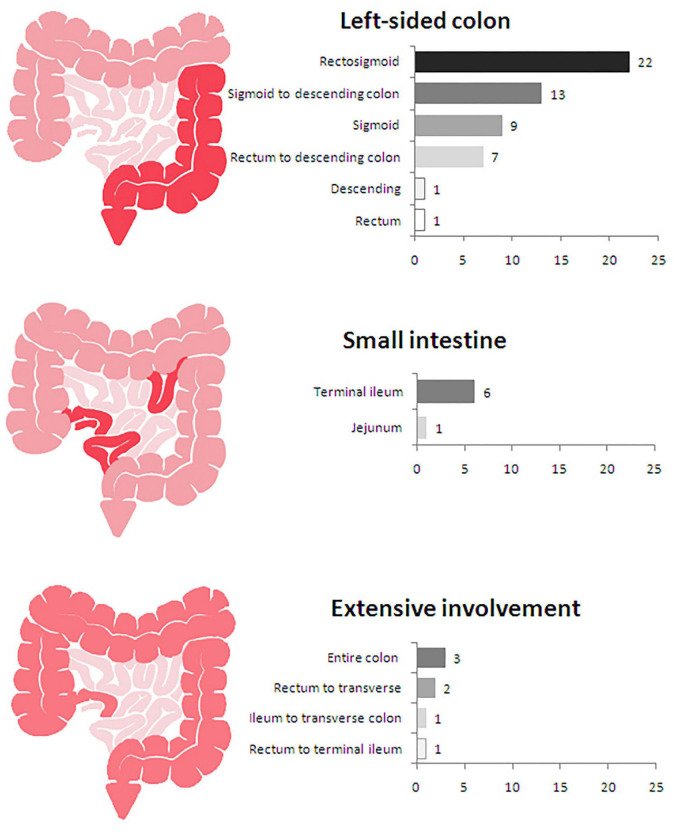
Locations of lesions in 70 patients with idiopathic myointimal hyperplasia of the mesenteric veins (IMHMV).

The most common bowel segment affected by IMHMV was the left colon. Intestinal involvement in decrescent order included the rectosigmoid (31.4%,22/70), sigmoid to the descending colon (18.6%, 13/70), sigmoid colon (12.9%,9/70), rectum to the descending colon (10%, 7/70), small intestine (10%, 7/70), pancolonic (4.3%, 3/70), rectum to the distal transverse (2.9%,2/70), ileum to transverse colon (1.4%,1/70), rectum(1.4%,1/70), descending colon (1.4%,1/70) and transverse colon (1.4%,1/70). The small intestine was affected in seven cases, including one case in the jejunum and six in the terminal ileum. Recently, an extremely rare case of IMHMV was reported with extensive involvement from rectum to the small intestine (30). The patient was operated several times due to anastomotic leakages and perforations and a duodenostomy was performed. Unfortunately, the patient died in the waiting period of a small bowel transplantation (Figure 3).

Clinical presentation varied between disease affecting the small intestine and the colon. With regard to colonic IMHMV, abdominal pain, hematochezia, and diarrhea were more common, similarly to what is seen in IBD or ischemic colitis; On the contrary, obstruction was more frequently seen in cases with ileum involvement. The meantime between symptom onset and surgery was 4.5 months (Table 1).

**TABLE 1:** Clinical characteristics of all reported cases of IMHMV to date.

	Authors, year	Age (y)/Sex	Affected Site	Clinical Impression	Indication for surgery	Time to surgery	Follow-up
1	Current case	64/M	Ileum	IBD	Bowel obstruction	6 months	1 yr
2	López Morales et al. (30)	37/M	Rectum to terminal ileum	CD	Abdominal pain	7 months	Died
3	Shah et al. (36)	24/F	Rectum to descending colon	IC	Abdominal pain/perforation	–	–
4–15	Kim et al. (37)	Mean 66(range 58–77)/11M&1F	Rectosigmoid (*n* = 9), rectum to descending colon (*n* = 2), ileum to transverse colon (*n* = 1)	IC (*n* = 4); UC (*n* = 1), non-specific colitis (*n* = 1), CMV colitis (*n* = 1), Idiopathic Phlebosclero colitis (*n* = 1), IMHMN (*n* = 3)	–	Mean 3 months (range 1–8 months)	Mean 29 months (range 2–125 months)
16	Wong et al. (29)	72/M	Sigmoid to descending colon	IC	Abdominal pain	–	–
17	Xie and Xu (28)	21/F	Rectosigmoid	IBD	Massive hematochezia	20 days	2 yr
18	Ansari et al. (25)	63/M	Sigmoid to descending colon	Entameba histolytica infection	Abdominal pain	>2 months	5 yr
19	Fang et al. (27)	21/F	Rectosigmoid	IBD	Hematochezia and perforation	2 months	1 yr
20	Yamada et al. (33)	81/F	Terminal ileum	Adhesive intestinal obstruction	Bowel obstruction	–	32 mo
21	Almumtin et al. (11)	55/M	Rectum to distal transverse	IBD	Perforation	1 yr	–
22	Wu et al. (34)	53/M	Rectum to descending colon	UC	Persisting symptoms	3 months	3 months
23	Martin et al. (26)	63/M	Sigmoid to descending colon	IC/IBD	Persisting symptoms	5 months	2 months
24	Chudy-Onwugaje et al. (32)	54/M	Transverse colon	CMV colitis	Persisting symptoms	4 months	–
25–32	Anderson et al. (35)	Median 62.5 (range 22–75)/6M&2F	Sigmoid (*n* = 6)	IBD (*n* = 3)	–	–	–
33	Louie et al. (17)	57/M	Small bowel	–	Abdominal pain	–	–
34	Gonai et al. (38)	68/M	Sigmoid to descending colon	mesenteric panniculitis	Persisting symptoms	–	–
35–44	Yantiss et al. (22)	Mean 68 (range 25–83)/9M&1F	Sigmoid to descending colon (*n* = 7), descending colon (*n* = 1), sigmoid colon (*n* = 1)	IC/IBD (*n* = 1), IBD (n = 7); IC (*n* = 2)	Perforation (*n* = 5), obstruction and refractory colitic symptoms	–	–
45	Song and Shroff (16)	59/M	Sigmoid to ileum	CD	Persisting symptoms	30 yr	2 wk
46	Yang et al. (39)	44/M	Rectosigmoid	UC	Persisting symptoms	4 wk	–
47	Patel et al. (40)	65/M	Sigmoid to descending colon	–	Perforation	1.5 months	–
48	Guadagno et al. (41)	59/F	Ileum	CD	Multiple ileal neuroendocrine tumors	6 months	3 months
49	Costa et al. (42)	47/M	Rectosigmoid	IC/IBD	Persistent symptoms	9 months	–
50	Cauchois et al. (10)	48/M	Rectum	IBD	–	3 months	–
51	Yun et al. (9)	64/M	Rectum to distal transverse	UC	Hematochezia	2 yr	6 months
52	Wangensteen et al. (8)	62/F	Rectosigmoid	UC	Persistent symptoms	2 months	1.5 yr
53	Abbott et al. (7)	58/M	Rectum to descending colon	IC/IBD	Persistent symptoms	–	–
54	Sahara et al. (43)	76/M	Rectosigmoid	IC/IBD	Persistent symptoms	1 yr	3 months
55	Laskaratos et al. (6)	62/F	Ileum	IBD	Perforation and hematochezia	–	–
56	Zijlstra et al. (5)	62/M	Rectum to descending colon	–	Acute abdomen	–	2 yr
57	Korenblit et al. (4)	59/M	Rectosigmoid	IC	Persistent symptoms	1 months	3 months
58	Feo et al. (44)	75/F	Rectosigmoid	IC	Persistent symptoms	6 months	–
59	Lanitis et al. (45)	81/M	Terminal ileum	–	Appendiceal mucocoele and pseudomyxoma peritonei	6 months	–
60	Korenblit et al. (3)	62/M	Entire colon (rectal sparing)	UC	Hematochezia	18 months	–
61	Chiang et al. (46)	60/M	Rectosigmoid	UC	Persistent symptoms	2 months	4 months
62	Garcia-Castellanos et al. (47)	32/M	Rectum to descending colon	primary pneumatosis intestinalis	Abdominal pain and hemotochezia	3 months	24 months
63	Kao et al. (31)	38/M	Rectosigmoid	IBD	Perforation	5 months	18 months
64	Savoie and Abrams, (48)	22/M	Rectosigmoid	IBD	Abdominal pain and hemotochezia	–	10 months
65	Bryant, (49)	42/F	Jejunum	–	–	–	–
66	Abu-Alfa et al. (14)	58/M	Sigmoid	IC/IBD	Abdominal pain and hemotochezia	1 yr	–
67–70	Genta and Haggitt, (1)	Mean 40 (range 25–67)/4M	Sigmoid (*n* = 1), Sigmoid to descending colon (*n* = 1), Rectosigmoid (*n* = 2)	UC (*n* = 2), CD (*n* = 1); Stricture (*n* = 1)	Bowel obstruction (*n* = 1), toxic megacolon (*n* = 1), abdominal pain and hemotochezia (*n* = 2)	Mean 3 months (range 1–6 months)	Mean 3.5 yr (range 1–7 yr)

*IC, ischemic colitis; IBD, inflammatory bowel disease; CD, Crohn’s disease; IC, ischemic colitis; CMV, cytomegalovirus; IMHMV, idiopathic myointimal hyperplasia of the mesenteric vein.*

### Endoscopic Characteristics

The endoscopic findings of IMHMV are a result of ischemic changes of the mucosa due to vasculopathy and the endoscopic appearance is often non-specific, contributing to the high rate of misdiagnosis. We reviewed 70 reported cases with IMHMV, of these, endoscopy was performed in 92.9% (65/70) of the cases. The most common endoscopic features were ulceration (69.2%, 45/65), mucosal congestion and friability (35.4%, 23/65), and stricture (13.8%, 9/65). A case series study revealed that diffuse mucosal erythema and friability affected the distal colorectum in all patients. In this study, extensive ulcers were present in 8 (89%) patients, and 6 (67%) had endoscopically apparent stricture (22).

At the early diseasestage, edematous, erythematous, and friable colonic mucosa may be seen, while ulceration and inflammatory exudates develop later with disease progression (11, 31). A case of mucosal edematous and erythematous lesions at onset was reported that progressed to more severe patchy ulcerations and inflammatory exudates within several weeks (31). A recent case study also described a patient with endoscopic findings of mild inflammation and congestion of the colon on admission which progressed to severely inflamed mucosa with significant narrowing of the lumen 2 weeks later (11). This indicates that the obvious mucosal changes may lag behind the onset of clinical symptoms, which manifest as a mild pattern at the early stage of the diseaseand that ulcers or luminal stricture may appear rapidly in weeks depending on the severity of vascular stenosis.

Various intestinal ulcers were documented in 45 reported cases of IMHMV, including irregular, circumferential, cobblestone, or cratered ulcers. Most intestinal ulcers were non-specific and suggestive of ischemia or idiopathic IBD (66.7%,30/45). These atypical ulcers may remind clinicians of the importance of diagnosing non-IBD preoperatively. Circumferential ulcers were described in 20% (9/45) of cases that mimicked ischemic colitis; however, it is difficult to distinguish them from other causes of ischemic colitis (22, 25). Longitudinal ulcers and cobblestone appearance were also seen in five cases of IMHMV, including our case, which are more suggestive of CD. Cratered ulcers were described in one case. The discrepancy between clinical and endoscopic findings in IMHMV has led to the clinical mismanagement of most reported cases of IMHMV as IBD (32).

### Radiological Features

Computed tomography (CT) of the abdomen with intravenous contrast is very useful for assessing the range of intestinal lesions in patients with IMHMV. Typical findings of IMHMV on CT include a segment of diffuse circumferential colonic wall thickening with submucosal edema, poor mural enhancement, and pericolic fat stranding, which are consistent with the diagnostic features of ischemic colitis (9, 11). There are rich dilated and winding peripheral veins surrounding the affected lesions. In angiographic imaging, these collateral circulations manifest as small, aneurysm-like lesions and are considered a compensatory mechanism for mesenteric venous ischemia. These radiologic features are helpful in the diagnosis of IMHMV (9, 27, 33).

Although IMHMV is often misdiagnosed as IBD, there are distinguishing imaging features that may aid in the preoperative diagnosis of this disease. One of the previous studies investigated the angiographic features of IMHMV and reported complete occlusion of the distal inferior mesenteric vein with peripheral venous ectasia on inferior mesenteric angiography (9). The finding of distal non-visualization of the inferior mesenteric vein could differentiate IMHMV from other bowel diseases such as ulcerative colitis; the latter share a similar CT appearance with IMHMV, including diffuse colonic mural thickening and enhancement and pericolic fat stranding, but have a patent inferior mesenteric vein (9). Moreover, CD commonly manifests with eccentric thickening in the mesenteric side with uneven reinforcement due to intestinal fibrosis and congestion of mesenteric vessels, which is termed as the “comb sign.” Additionally, unlike CD, which usually presents the typical “skip sign,” IMHMV lesions are continuously distributed through the intestinal segment (Table 2).

**TABLE 2:** Comparison between IMHMV and CD.

	IMHMV	CD
Onset age	Older (mean age 58 years old)	Younger (18–35 years old)
Clinical features	Abdominal pain>hemotochezia>diarrhea, complicated with intestinal bleeding and perforation	Diarrhea>abdominal pain>weight loss, perianal involvement and extraintestinal manifestations are common
Sites of involvement	Rectosigmoid and descending colon, rarely in small intestine	Terminal ileum and ileocecum>colon>rectum>small intestine>upper digestive tract
Skipped lesions	No	Yes
Ulcers	Non-specific ulcers	Longitudinal ulcers, cobblestone appearance and aphthous ulcers
Histopathology	Intima and media smooth muscle proliferation	Non-caseating granuloma
Treatment	Surgery, no response to medication	Response to 5-Aminosalicylic acid, steroid, immunosuppressant or biologic agents
Recurrence post operation	No	Yes

*CD, Crohn’s disease; IMHMV, idiopathic myointimal hyperplasia of the mesenteric vein.*

Compared with colonic IMHMV, small bowel IMHMVshows different features. Luminal stenosis is frequent in the small bowel, but no obvious diffuse circumferential wall thickening was observed in colonic lesions (33). One possible explanation might be the smaller luminal diameter of the ileum. Accordingly, the inflammation seen in IMHMV might more strongly influence the change in the caliber of the small intestine, resulting in bowel obstruction (34). In the present case, a chrysanthemum-like change was found on CT enterography, suggesting the possibility of an intestinal fistula similarly to what is seen in CD. To the best of our knowledge, this is the first case report of chronic ileum perforation of IMHMV, which helps increase our knowledge of this disease.

### Treatment

Surgical resection is the mainstay of therapy for IMHMV and postoperative recurrence of the disease has not been described in follow-up duration up to 7 years after resection. It has been reported that IMHMV is refractory to medical treatment (35), however, majority of the cases were first treated with IBD-directed medical management (including corticosteroids, 5-aminosalicylates, immunomodulators, or biologic agents). Early diagnosis may decrease misdiagnosis of IBD and decrease patients’ exposure to unnecessary IBD treatment.

**FIGURE 4:**
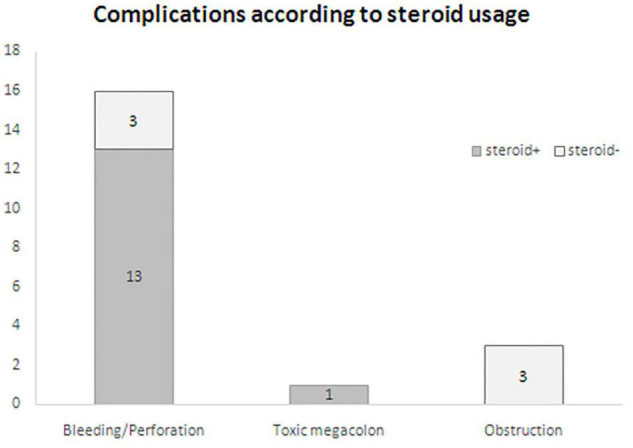
Complications according to steroid usage.

After reviewing the 70 cases of IMHMV described in the literature, complications of IMHMV occurred in 20 amount of cases. Complications were mainly attributed to IBD-related medication (14/20, 8 with bowel perforation, 3 with massive hematochezia, 2 with both bowel perforation and hematochezia, and 1 with toxic megacolon), delay of operation, and need for emergent surgery (60%, 12/20). Of these, intestinal perforation and/or bleeding were the most common complications, accounting for 80% (16/20) of cases. Interestingly, a higher proportion of patients with intestinal perforation and/or bleeding were observed after steroid use (81.3%, 13/16) than among those not using steroids (18.8%, 3/16) (Figure 4). Moreover, all reported cases of intestinal perforation had an acute onset, which was different from our case of small bowel obstruction with a chronic course and fistula formation.

## Conclusion

Idiopathic myointimal hyperplasia of the mesenteric veins is a rare condition that leads to chronic colonic ischemia. It should be suspected in middle-aged patients with sub-acute segmental enteritis with clinical findings suggestive of IBD or chronic intestinal ischemia, especially in those refractory to medical treatment; however, preoperative diagnosis is challenging. The resection is curative, and there is no known evidence of disease recurrence.

This is a comprehensive literature search with the latest published case reports to date. In this review, the clinical features of IMHMV were analyzed, and the difference between IMHMV and Crohn’s disease was compared, which may raise clinician’s awareness of this disease and avoid inappropriate treatment. The findings of the present study are limited, nevertheless, by the quality and integrity of the data in the case reports, and the true incidence of IMHMV is likely to be underestimated due to the lack of recognition.

## Author Contributions

HL and FT designed the structure of the manuscript and wrote the manuscript. HS and YG analyzed the pathological and imaging data. MC and HZ drafted the manuscript. HZ and FT revised the manuscript. All authors read and approved the final manuscript.

## Conflict of Interest

The authors declare that the research was conducted in the absence of any commercial or financial relationships that could be construed as a potential conflict of interest.

## Publisher’s Note

All claims expressed in this article are solely those of the authors and do not necessarily represent those of their affiliated organizations, or those of the publisher, the editors and the reviewers. Any product that may be evaluated in this article, or claim that may be made by its manufacturer, is not guaranteed or endorsed by the publisher.

## Abbreviations

AVF: arteriovenous fistula
CD: Crohn’s disease
CT: computed tomography
ELP: enterocolic lymphocytic phlebitis
IMHMV: idiopathic myointimal hyperplasia of the mesenteric veins
IBD: inflammatory bowel disease.
